# Microglial process convergence onto injured axonal swellings, a human postmortem brain tissue study

**DOI:** 10.21203/rs.3.rs-4713316/v1

**Published:** 2024-08-09

**Authors:** Amanda L. Logan-Wesley, Karen M. Gorse, Audrey D. Lafrenaye

**Affiliations:** Virginia Commonwealth University; Virginia Commonwealth University; Virginia Commonwealth University

**Keywords:** Microglial process convergence, Traumatic brain injury, Axonal injury, postmortem tissue

## Abstract

Traumatic brain injury (TBI) affects millions globally, with a majority of TBI cases being classified as mild, in which diffuse pathologies prevail. Two of the pathological hallmarks of TBI are diffuse axonal injury and microglial activation. While progress has been made investigating the breadth of TBI-induced axonal injury and microglial changes in rodents, the neuroinflammatory progression and interaction between microglia and injured axons following brain injury in humans is less well understood. Our group previously investigated microglial process convergence (MPC), in which processes of non-phagocytic microglia directly contact injured proximal axonal segments, in rats and micropigs acutely following TBI. These studies demonstrated that MPC occurred on injured axons in the micropig, but not in the rat, following diffuse TBI. While it has been shown that microglia co-exist and interact with injured axons in humans post-TBI, the occurrence of MPC has not been quantitatively measured in the human brain. Therefore, in the current study we sought to validate our pig findings in human postmortem tissue. We investigated MPC onto injured axonal swellings and intact myelinated fibers in cases from individuals that sustained a TBI and control human brain tissue using multiplex immunofluorescent histochemistry. We found an increase in MPC onto injured axonal swellings, consistent with our previous findings in micropigs, indicating that MPC is a clinically relevant phenomenon that warrants further investigation.

## Introduction

Traumatic brain injury (TBI) affects an estimated sixty-nine million people globally each year[1]. In 2022 alone, over twenty thousand United States service members from the Army, Navy, Air Force, and Marines, suffered from a TBI[2]. Approximately 80% of all TBI cases are classified as mild, in which diffuse pathologies that are difficult to discern via molecular imaging prevail. One of the pathological hallmarks of mild TBI is diffuse axonal injury, wherein axons are disrupted over time and progress to disconnection resulting in a proximal axonal swelling that is still connected to the neuronal soma and a distal axonal segment that degenerates via Wallerian degradation[3]. Additionally, microglia, the innate immune cell of the central nervous system, have been shown to be activated following TBI in both humans[4–9] and animals[10–14] and have been linked to cognitive changes following TBI[5]. Activated microglia fall on a spectrum from pro-inflammatory to anti-inflammatory with functions that can promote tissue neurodegeneration or neuroprotection[15–21].

While previous studies have identified various pro and anti-inflammatory pathways upregulated following TBI, non-phagocytic physical interactions between activated microglia and adjacent neurons have only recently begun to be investigated[10, 22–29]. Previous studies from our group using a micropig model of TBI found microglial processes converging onto the injured proximal axonal segment in a phenomenon called microglial process convergence (MPC)[27, 30]. This MPC does not appear to involve phagocytosis[27] and was not found in our rat model of TBI[26]. Specifically, in pigs, the number of activated microglial processes contacting injured proximal axonal swellings was nearly twice that observed for non-injured myelinated fibers at 6hrs following a diffuse TBI generated using the central fluid percussion injury model[27]. This MPC significantly increased from 6hrs to 1 day post injury[26].

However, in rats, there were far fewer microglial processes contacting injured axonal swellings compared to non-injured myelinated fibers following TBI, indicating that MPC might be a phenomenon associated with higher order gyrencephalic brains[26]. To investigate the potential that TBI-induced MPC onto injured axons occurs in the human brain, in the current study we quantitatively assessed the prevalence of MPC onto injured axonal swellings and intact axonal segments in human postmortem brain tissue.

## Results

### Microglial Process Convergence Increases Following Traumatic Brain Injury and Axonal Injury

**Figure 1:**
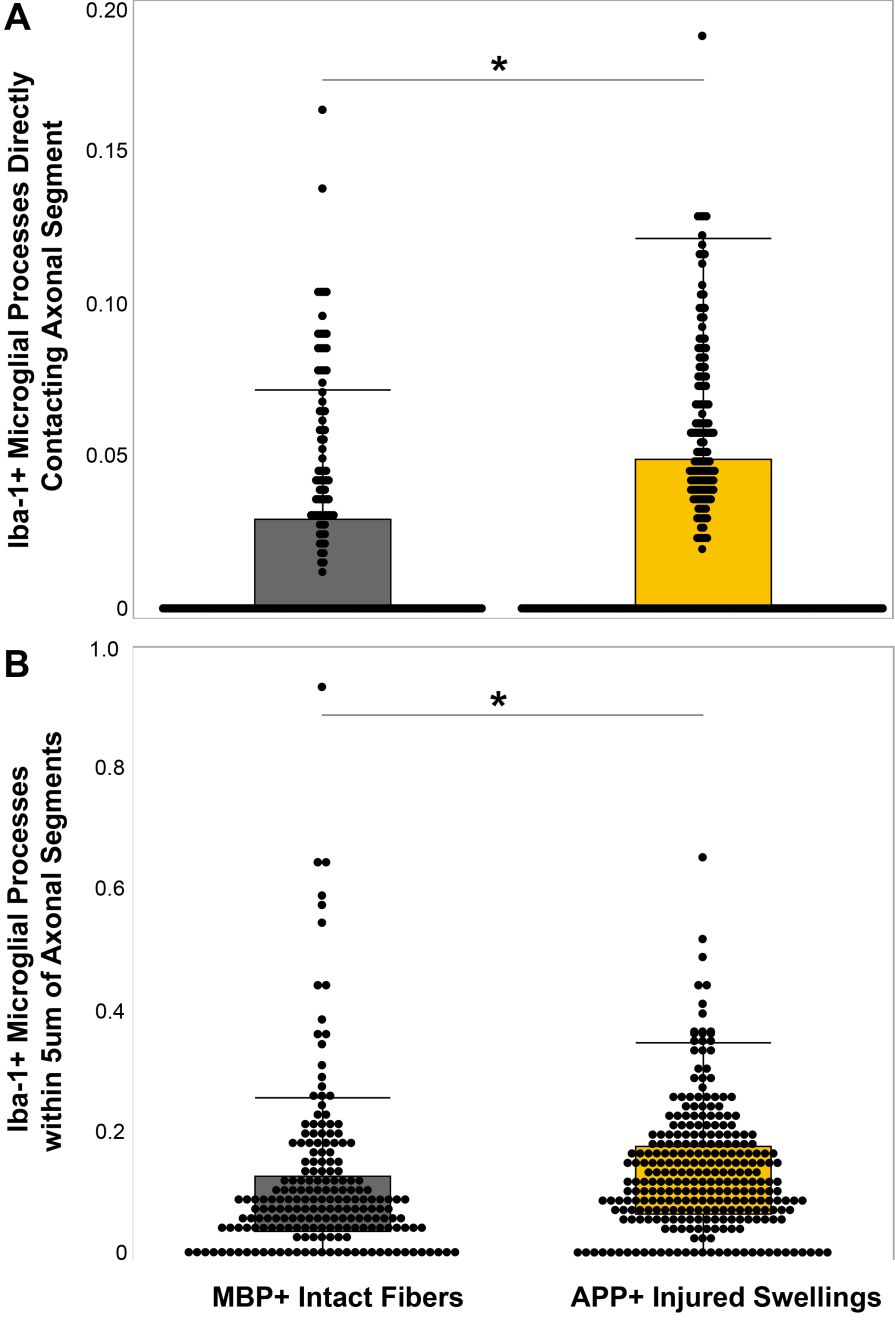
Microglial processes converged onto injured axons in human postmortem tissue. Box and whisker plots of Iba-1+ microglial processes A) in direct contact with or B) within 5mm of MBP+ non-injured intact axonal fibers (n=197) and APP+ injured axonal swellings (n=278). * p<0.05.

To investigate the potential for microglial process convergence occurring on injured axonal swellings or intact myelinated fibers in the human brain, multiplexed immunohistochemistry against APP to visualize injured axons, MBP to visualize intact myelinated axons, and Iba-1 to visualize microglia processes was done on human postmortem tissue from the DoD/USU tissue repository. When all fibers that were analyzed across all cases were collated as APP + injured axonal swelling or MBP + intact myelinated fibers, it was found that more Iba-1 + microglial processes/um of the perimeter were in direct contact with APP + axonal swellings compared to MBP + intact myelinated fibers (Fig. 1; U = 32,240, *p* = 2.4×10^−4^). The paraffin sections were ~ 5μm thick, precluding the ability to perform 3D reconstructions of the axonal swellings, as we had done for our previous studies[26, 27]. As a single 2D image of the axonal segments is likely to be missing processes that are out of the plan of section, we also investigated the number of Iba-1 + microglial processes that were within 5um of the axonal segments. More microglial processes were found within 5um of APP + axonal swellings compared to MBP + intact myelinated fibers (Fig. 1; U = 34,260, *p* = 2.99×10^−6^), indicating that more microglial processes are close to the injured axonal swellings.

**Figure 2:**
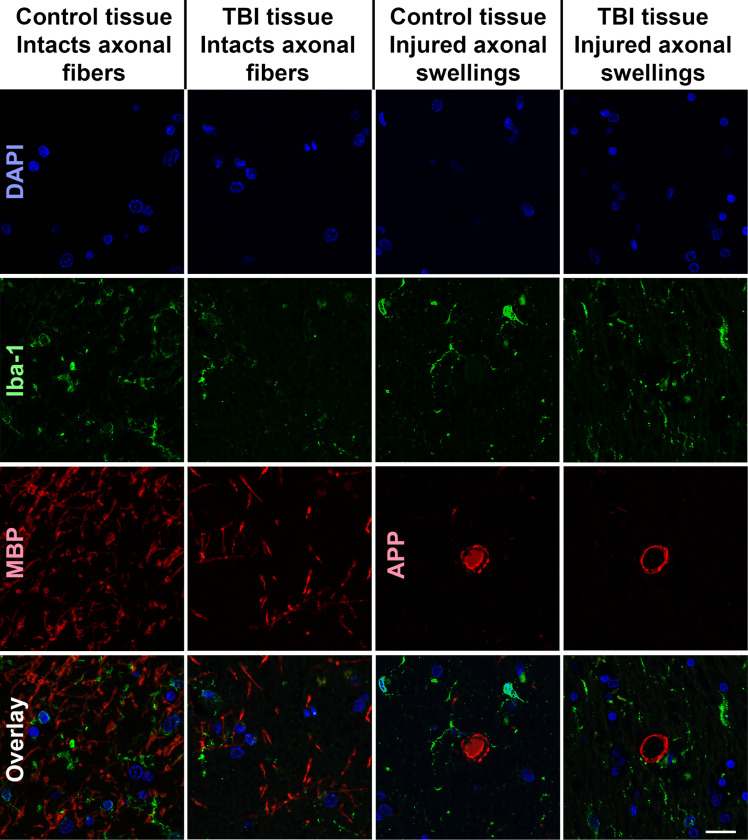
Representative fluorescent micrographs of microglial process interactions with either non-injured intact myelinated axonal fibers (first two columns) or injured axonal swellings (second two columns). Nuclei were labeled with Dapi in blue (top panel). Microglia were immunolabeled with Iba-1 which is pseudo colored green in the second panel. Intact myelinated fibers immunolabeled with MBP (first two columns of the third panel) or injured axonal swellings immunolabeled with APP (last two columns of the third panel) were pseudo colored in red. The last panel shows the full overlay for the multiplex immunohistochemical labeling. Scale bar is 20mm.

**Figure 3:**
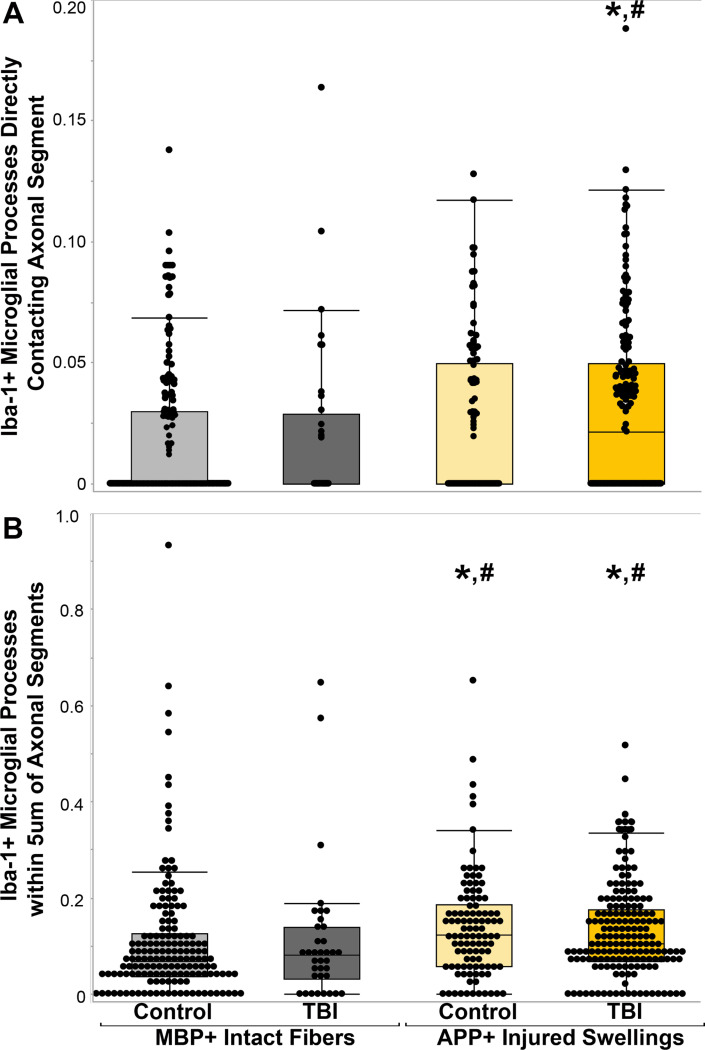
Microglial processes appear to converge onto injured axons in control and TBI cases. Box and whisker plots of Iba-1+ microglial processes A) in direct contact with or B) within 5mm of MBP+ non-injured myelinated axonal fibers in either control cases (n=161 fibers) or TBI cases (n=36 fibers) and APP+ injured axonal swellings in control cases (n=105 swellings) or TBI cases (n=173 swellings). Note that while the APP+ axonal swellings within the control cases did not have significantly higher Iba-1+ microglial processes directly touching it (p=0.053), both control cases and TBI cases had significantly more Iba-1+ microglial processes within 5mm compared to the non-injured MBP+ fiber counterparts. * p<0.05 compared to MBP+ intact fibers in control tissue. # p<0.05 compared to MBP+ intact fibers in control tissue.

After completing this initial analysis of the overall comparison between APP + swellings and MBP + myelinated fibers, the cases were un-blinded. Following case unblinding, it was discovered that some APP + axonal swellings were identified in control individuals and some MBP + myelinated fibers were analyzed from individuals that suffered a TBI (Fig. 2). Therefore, the analyzed fibers were organized into four groups: 1) MBP + intact myelinated fibers in control tissue, 2) MBP + intact myelinated fibers in TBI tissue, 3) APP + axonal swellings in control tissue, and 4) APP + axonal swellings in TBI tissue. When the data was stratified by axonal injury and TBI the finding of more microglial process convergence occurring directly onto APP + injured axonal swellings, specifically within TBI tissue (χ^2^(3) = 15.53, *p* = 0.001; Fig. 3). There were also significantly more microglia processes within 5μm of APP + injured swellings in both control and TBI tissue as compared to MBP + fibers in either injured or control postmortem samples was maintained (χ^2^(3) = 21.97, *p* = 6.61×10^−5^; Fig. 3).

**Figure 4:**
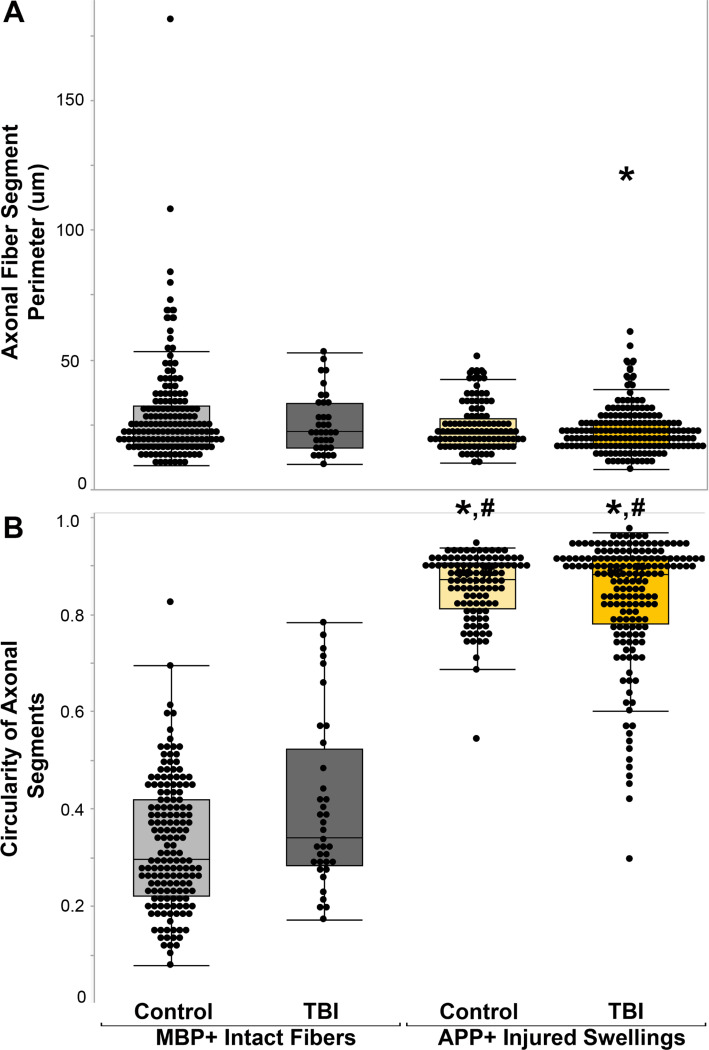
Injured axons have lower perimeters and are more circular than MBP+ intact myelinated fibers. Box and whisker plots of A) the perimeter of axonal segments and B) the circularity of axonal segments of MBP+ intact axonal fibers in either control cases (n=161 fibers) or TBI cases (n=36 fibers) and APP+ injured axonal swellings in control cases (n=105 swellings) or TBI cases (n=173 swellings). Note that while there was no significant difference between APP+ axonal swellings in control tissue compared to MBP+ myelinated fibers in control tissue (p=0.08), injured APP+ axonal swellings within TBI tissue had significantly lower perimeters. * p<0.05 compared to MBP+ intact fibers in control tissue. # p<0.05 compared to MBP+ intact fibers in control tissue.

The morphology between MBP + intact axonal segments and APP + axonal swellings was significantly different (χ^2^(3) = 12.2, p = 0.007), with the APP + axonal swellings within TBI tissue having smaller perimeters compared to the MBP + intact axonal fibers within control tissue (p = 0.003). The APP + axonal swellings in both control and TBI tissue had significantly higher circularity indices compared to the MBP + axonal fibers in either control or TBI tissue (χ^2^(3) = 332.35, p = < 0.001; Fig. 4). While the MBP + intact axonal fibers within TBI tissue were more circular than the MBP + axonal fibers in control tissue (p < 0.001), the morphology of APP + axonal swellings within control cases, however, were consistent with the APP + axonal swellings in TBI cases (Fig. 4; Perimeter p = 0.08; circularity p = 0.83).

**Figure 5:**
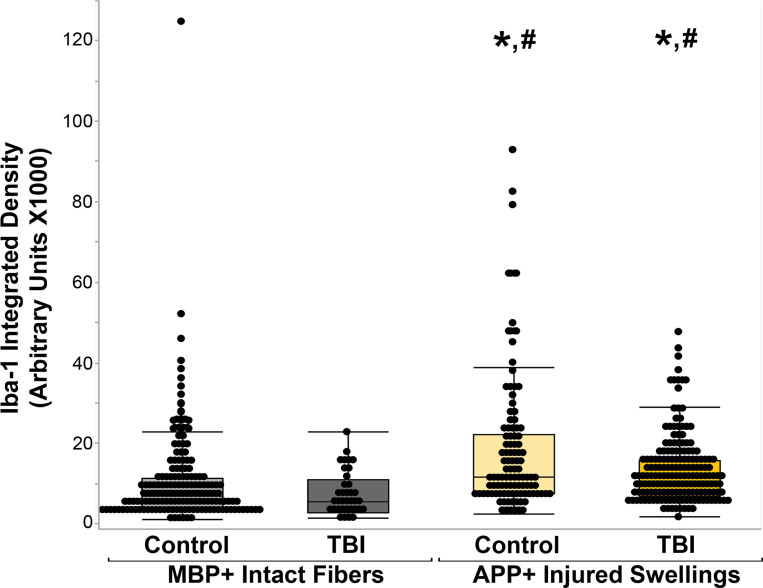
Injured axons have higher Iba-1+ microglia fluorescent intensity than MBP+ intact myelinated fibers. Box and whisker plots depicting the integrated density of Iba-1+ microglia within axonal segments of MBP+ intact axonal fibers in either control cases (n=161 fibers) or TBI cases (n=36 fibers) and APP+ injured axonal swellings in control cases (n=105 swellings) or TBI cases (n=173 swellings). Injured APP+ axonal swellings in either control or TBI tissue had significantly higher Iba-1+ fluorescent intensity within the swellings compared to intact MBP+ axonal fibers. * p<0.05 compared to MBP+ intact fibers in control tissue. # p<0.05 compared to MBP+ intact fibers in control tissue.

To validate the by eye counts of Iba-1 microglial process puncta onto the APP + axonal swellings, the integrated density/intensity of Iba-1 microglial processes in the region of the APP + axonal swelling or MBP + myelinated fiber was assessed. The intensity of Iba-1 labeling was significantly higher in APP + injured axonal swellings compared to control MBP + axons (χ^2^(3) = 56.217, *p* = 3.78×10^−12^; Fig. 5). Specifically, there were not differences in the intensity of Iba-1 between APP + injured axons (p = 0.72) nor between MBP + axonal segments (p = 0.99) within control tissue compared to TBI tissue. There was significantly higher Iba-1 integrated density within APP + axonal swellings compared to MBP + intact axonal segments regardless of TBI status of the case (MBP + intact fiber in control cases vs. APP + swellings in control cases p < 0.001; MBP + intact fiber in TBI cases vs. APP + swellings in TBI cases p < 0.001; Fig. 5). This finding validated the counts of microglial process convergence onto injured axonal swelling, demonstrating increases in microglial process convergence onto APP + injured axonal swellings in the human brain.

## Discussion

The current study demonstrates that microglial processes converge onto injured axonal swellings in the human brain following TBI. The perimeters of the axonal segments were relatively consistent across non-injured MBP + myelinated fibers in both control and TBI tissue as well as APP + injured axonal swellings within control tissue. The APP + injured axonal swellings within TBI tissue had significantly lower perimeters than the MBP + intact myelinated axonal segments within control tissue. The number of Iba-1 + microglial processes in direct contact with the axonal segment, however, was significantly higher onto APP + axonal swellings compared to MBP + non-injured axonal segments, despite the lower perimeter available for contact. There were also significantly more microglial processes within 5μm of the injured axons compared to the non-injured MBP + axonal segments. Our group previously observed MPC in the micropig brain acutely following a central fluid percussion diffuse TBI[27]. This phenomenon; however, was not recapitulated following TBI in the rat[26], indicating that MPC onto injured axons might be species specific. The current findings that MPC onto injured axonal swellings occurs in human postmortem tissue, validates our previous findings in the micro pig and indicates that MPC following TBI might primarily manifest in higher order gyrencephalic brains. These findings show that MPC is a phenomenon that occurs in the human population, necessitating further investigation.

Diffuse axonal injury (DAI) or traumatic axonal injury (TAI) is one of the hallmark pathologies of mild TBI[31–36]. It was originally thought that DAI occurs following TBI due to the mechanical shearing of the axons. However, this is only true for a subset of axons that are observed to be injured within minutes of TBI, which is referred to as primary axotomy. Rather, secondary axotomy/axonal injury, which occurs sub-acutely after injury in rodent models of TBI is the phenomenon that is typically investigated. Secondary axonal injury involves accumulation of the cytoskeleton and organelles within the axons[37–39]. Specifically, the tensile forces of the TBI cause axonal alterations that allow an influx of calcium into the axon. This calcium influx leads to activation of cysteine protease pathways, which leads to degradation of the cytoskeleton and ultimately lead to a reactive axonal swelling as anterogradely transported proteins and organelles pool at the end of the proximal axon[38–40]. In the early 1990s immunohistochemistry against the anterogradely transported protein, APP, was found to efficiently label the proximal injured axonal segment where it pooled[41, 42]. Immunohistochemical labeling of APP has since become the gold standard for identifying DAI pathologically. Secondary axonal injury is typically studied in rodents 6–24 hours following injury, when it is most prevalent[43, 44]. Within higher order animals DAI appears to be prevalent starting hours following injury and peaking at 1w post-TBI[13], however DAI has been shown to last up to 6 months in a pig model of TBI[45] and has been observed in human postmortem tissue from people several years following a TBI[46, 47].

Many recent studies have demonstrated the impact of inflammatory cascades in regulating behavioral morbidities, general pathology, and neuronal function in both the normal brain and in various disease states, including TBI[21, 48, 49]. Neuroinflammation has been demonstrated in various brain regions in the human population chronically following TBI[5, 9, 20, 50]. Microglia, the innate immune cells of the brain, are critical mediators of these TBI-induced neuroinflammatory processes[16, 51–57]. Microglia have been shown to contact specific areas of the axon in the mouse brain during homeostasis including, the nodes of Ranvier[58], the axon initial segment[10], synapses[59], and neuronal soma[60]. Many studies using rodents have indicated that reduction or elimination of activated microglia and/or targeting various neuroinflammatory signaling pathways ameliorates downstream pathology and behavioral morbidity[24, 61–64]. Conversely, other studies have also found that anti-inflammatory microglial activation is necessary and potentially advantageous[17, 54, 65–70]. These studies demonstrated activated microglia can secrete neurotrophic factors[15, 71–73], which would suggest a potential ameliorative effect of microglia following injury in some cases. Additionally, recent studies have shown that microglia physical contacts play a role in regulating neuronal activity, either increasing activity following anesthesia[74, 75] or decreasing activity following epileptiform activity[60, 67].

A study by Schirmer et. al. investigating human post-mortem tissue found a significant positive correlation between the density of microglia and axonal outgrowth as well as the duration of patient survival following TBI, however, they did not quantitatively investigate the physical interactions between microglia and injured axons[76]. Another study done in vitro and in rats following a spinal cord injury found that exosomes from anti-inflammatory microglia increased neurite outgrowth in vitro and increased GAP43 expression in vivo, indicating that microglia could play a role in axonal outgrowth[17].

Microglia have been observed within physical proximity to injured axonal swellings in human postmortem tissue[76–78]. Oehmichen et al. observed an increase in CD-68 + microglial cells areas of axonal injury in the white matter at least five days post-TBI in human postmortem tissue, however, they only observed limited physical interactions between the CD68 + cells and the APP + axonal segments[77]. Ryu et al. qualitatively identified areas in which Iba-1 + microglia were in proximity to APP + injured axons in postmortem tissue from individuals following both motor vehicle accident and blast induced TBI[78]. Although, these previous studies indicated that there could be direct physical interactions between microglia and injured axonal segments, our current study is the first to quantitatively show that MPC occurs onto injured proximal axonal segments in human brain tissue following TBI.

We do appreciate that there are limitations to the current study, mainly, that all cases were from male doners. There is evidence that males and females respond to TBI differently[79]. A recent study also found that the burden of axonal injury following a diffuse TBI in a pig model was significantly higher in females compared to age matched males[80]. Further, microglia have been shown to be different in males and females[81–85]. Therefore, investigations into MPC in both the male and female population should be done to fully appreciate the prevalence of MPC onto injury axons in the human brain. Additionally, these sections were only 15μm thick, precluding a 3D investigation of MPC, as was done in our previous micro pig studies[26, 27]. It is likely that the numbers of microglial processes we found converging onto the axonal segments in the current study were artificially lower than they might actually be due to the section thickness. Therefore, investigations using thicker tissue in which 3D reconstructions could be done, would be warranted to glean a better appreciation of the degree of MPC onto injured axonal swellings in human postmortem tissue of both males and females. Despite these limitations, the current study is the first to quantitatively demonstrate MPC onto injured axons in the human brain following TBI. These findings indicate that MPC is a component of human TBI and that further studies exploring the phenotypes and overall roles of the microglia involved in MPC following TBI should be investigated.

## Methods

### Samples

Human brain samples were acquired from the Department of Defense (DoD)/Uniform Services University (USU) Tissue Repository. All cases were from males between 26–69 years old (median age of 36 years) with a maximum postmortem interval of 1day. All identifiers were removed from the samples. Tissue was paraffin embedded and sectioned at 15um. Slides containing areas demonstrating axonal injury regardless of brain region were used for this study. A total of 11 human brain samples were used. Of these samples, 6 were confirmed TBI cases with demonstrated areas of axonal injury and 5 were controls. All study staff was blinded to case group throughout the labeling, imaging, and analysis.

### Immunohistochemistry

To visualize the interactions between microglia and injured or intact axonal segments in the human brain multiplexed fluorescent immunohistochemistry was performed. To identify injured axons, an antibody against amyloid precursor protein (APP) was used, which indicates axonal transport issues indicative of axonal injury [13, 14, 58]. To visualize microglia, an antibody against ionized calcium-binding adaptor molecule 1 (Iba-1) was used. Intact, non-injured axonal fibers were visualized using an antibody against myelin basic protein (MBP).

In this procedure, sections were deparaffinized by incubating slides in progressively more concentrated alcohols. Antigen retrieval was done by steaming the tissue in pH 6.0 citric acid buffer for 30min. Tissue was then blocked and permeabilized at room temperature in 5% normal goat serum (NGS), 2% bovine serum albumin (BSA) and 1.5% triton in phosphate buffered saline for 2hr followed by overnight incubation with a rabbit antibody against microglial Iba-1 (1:200; Cat.#019–19741 Wako; Richmond, VA, USA) at 4C° in 5% NGS/2% BSA/0.5% triton. Tissue was washed with 1%NGS/1%BSA in PBS at least six times prior to secondary antibody incubation with Alexa Fluor 488-conjugated goat anti-rabbit IgG (1:700; Cat.# A- 11008, Life Technologies, Carlsbad, CA, USA) in 1%NGS/1%BSA/PBS at room temperature for 2hr. Tissue was washed in PBS at least four times prior to overnight incubation with a mouse antibody against the 22C11 clone of APP (1:200; Cat.#14–9749-82, ThermoFisher Scientific, Waltham, MA, USA) in 5% NGS/2% BSA/0.5% triton at 4C°. Tissue was washed with 1%NGS/1%BSA in PBS at least six times prior to the next secondary antibody incubation with Alexa Fluor 647-conjugated goat anti-mouse IgG (1:700; Cat.# A- 21237, Life Technologies, Carlsbad, CA, USA) in 1%NGS/1%BSA/PBS at room temperature for 2hr. Iba-1 and APP labeled tissue was washed in PBS at least four times prior to overnight incubation with a rat antibody against MBP (1:200; Novus) at 4C° in 5% NGS/2% BSA/0.5% triton. Tissue was washed with 1%NGS/1%BSA in PBS at least six times prior to the third secondary antibody incubation with Alexa Fluor 568-conjugated goat anti-rat IgG (1:700; Cat.# A- 11077, Life Technologies, Carlsbad, CA, USA) in 1%NGS/1%BSA/PBS at room temperature for 2hr. Tissue was washed in PBS at least four times. Multiplex labeled tissue was coversliped with Vectashield hard-set mounting medium with Dapi (Cat.#H-1500; Vector Laboratories, Burlingame, CA, USA).

### Microglial Process Convergence Analysis

The fluorescently immunolabeled slides were imaged on the Keyence BZ-X800 microscope (Keyence Corporation of America, Itasca, IL, USA) at 40X magnification. One section was analyzed for each case by an investigator blinded to group. A navigation super-image was generated using the far-red channel in which the APP + injured axonal swellings could be visualized. Images containing at least 1 APP + injured axonal swellings were captured or no APP + swellings but clean MBP labeling were captured. Fewer MBP only images were captured, as there were several analyzable MBP + intact myelinated fibers in each captured image, whereas there were few analyzable APP + axonal swellings in each captured image. At least 25 images were taken for most cases, however, only 13 images were captured for 1 case as no APP + swellings were identified. Across all samples a total of 161 non-injured axonal segments from controls, 36 non-injured axonal segments from injured samples, 105 injured axonal swellings from controls, and 173 injured axonal swellings from injured samples were analyzed for the current study. Fiji Image J software (National Institute of Health, Bethesda, MD, USA) was used to evaluate the 2D images. Image scales were set to 5.3 pixels/um

To assess the interaction between microglia and injured axonal swellings, the APP + axonal swelling was traced using the freehand tool and measured for perimeter, area, shape descriptors (aspect ratio, circularity, round, solidity), integrated density, and mean grey value. The number of microglial processes and puncta that were directly touching the APP + axonal swelling was counted by hand. Then, the region encircling the APP + axonal swelling was enlarged by 5um and the microglial processes and puncta within the enlarged region was counted by hand.

In order to visualize the interaction between microglia and intact axonal segments, a random number generator was used to generate x,y coordinates to choose a MBP + axonal segment on the image. The axonal segment was traced with the freehand tool and measured for perimeter, area, shape descriptors (aspect ratio, circularity, roundness, and solidity), integrated density, and mean grey value. The number of microglial processes and puncta that were directly touching the MBP + intact axonal segment was counted by hand. Then, the region encircling the axonal segment was enlarged by 5um and the number of microglial processes and puncta within the enlarged region were counted by hand.

### Statistical Analysis

The statistics were run using IBM SPSS software (IBM Corp., Armonk, NY). A Shapiro-Wilk test was conducted to test for normality of the data. As the data was not normally distributed, a Mann-Whitney U test was used to test differences between all APP + injured axonal swellings and all MBP + intact axonal segments. A Kruskal-Wallis test was run to assess differences across multiple groups. A Bonferroni post hoc was used to correct for multiple pairwise comparisons. Statistical significance was set to a *p*-value of < 0.05. Data is presented as means and standard error of the mean. All raw data is included in Supplemental table 1.

## Data Availability

All data generated or analyzed during this study are included in this published article and its supplementary information files. Following publication these data will also be available on the Open Science Framework along with the analysis protocol.

## References

[1] Dewan MC, Rattani A, Gupta S, Baticulon RE, Hung Y-C, Punchak M, Estimating the global incidence of traumatic brain injury. Journal of Neurosurgery. 2018;130:1080–97. 29701556 10.3171/2017.10.JNS17352

[2] DOD TBI Worldwide Numbers [Internet]. Military Health System. [cited 2024 May 28]. Available from: https://www.health.mil/Military-Health-Topics/Centers-of-Excellence/Traumatic-Brain-Injury-Center-of-Excellence/DOD-TBI-Worldwide-Numbers

[3] Kelley BJ, Farkas O, Lifshitz J, Povlishock JT. Traumatic axonal injury in the perisomatic domain triggers ultrarapid secondary axotomy and Wallerian degeneration. Exp Neurol. 2006;198:350–60. 16448652 10.1016/j.expneurol.2005.12.017

[4] Coughlin JM, Yuchuanwang Y, Minn I, Bienko N, Ambinder EB, Xu X, Imaging of glial cell activation and white matter integrity in brains of active and recently retired national football league players. JAMA Neurology. 2017;74:67–74. 27893897 10.1001/jamaneurol.2016.3764 PMC5504689

[5] Coughlin JM, Wang Y, Munro CA, Ma S, Yue C, Chen S, Neuroinflammation and brain atrophy in former NFL players: An in vivo multimodal imaging pilot study. Neurobiology of Disease. 2015;74:58–65. 25447235 10.1016/j.nbd.2014.10.019 PMC4411636

[6] Little DM, Kraus MF, Joseph J, Geary EK, Susmaras T, Zhou XJ, Thalamic integrity underlies executive dysfunction in traumatic brain injury. Neurology. 2010;74:558–64. 20089945 10.1212/WNL.0b013e3181cff5d5 PMC2830915

[7] Boche D, Perry VH, Nicoll J a R. Review: Activation patterns of microglia and their identification in the human brain. Neuropathology and Applied Neurobiology. 2013;39:3–18. 23252647 10.1111/nan.12011

[8] Neumann KD, Seshadri V, Thompson XD, Broshek DK, Druzgal J, Massey JC, Microglial activation persists beyond clinical recovery following sport concussion in collegiate athletes. Front Neurol [Internet]. 2023 [cited 2024 May 1];14. Available from: 10.3389/fneur.2023.1127708/full PMC10080132 37034078

[9] Velázquez A, Ortega M, Rojas S, González-Oliván FJ, Rodríguez-Baeza A. Widespread microglial activation in patients deceased from traumatic brain injury. Brain Injury. 2015;29:1126–33. 26067626 10.3109/02699052.2015.1018325

[10] Baalman K, Marin M a., Ho TS-Y, Godoy M, Cherian L, Robertson C, Axon Initial Segment-Associated Microglia. Journal of Neuroscience. 2015;35:2283–92. 25653382 10.1523/JNEUROSCI.3751-14.2015 PMC4315845

[11] Cao T, Thomas TC, Ziebell JM, Pauly JR, Lifshitz J. Morphological and genetic activation of microglia after diffuse traumatic brain injury in the rat. Neuroscience. 2012;225:65–75. 22960311 10.1016/j.neuroscience.2012.08.058 PMC3489473

[12] Chhor V, Moretti R, Le Charpentier T, Sigaut S, Lebon S, Schwendimann L, Role of microglia in a mouse model of paediatric traumatic brain injury. Brain, Behavior, and Immunity. 2017;63:197–209. 27818218 10.1016/j.bbi.2016.11.001 PMC5441571

[13] Grovola MR, Paleologos N, Brown DP, Tran N, Wofford KL, Harris JP, Diverse changes in microglia morphology and axonal pathology during the course of 1 year after mild traumatic brain injury in pigs. 2021; 10.1111/bpa.12953 PMC8412066 33960556

[14] Witcher KG, Bray CE, Dziabis JE, McKim DB, Benner BN, Rowe RK, Traumatic brain injury-induced neuronal damage in the somatosensory cortex causes formation of rod-shaped microglia that promote astrogliosis and persistent neuroinflammation. Glia. 2018;66:2719–36. 30378170 10.1002/glia.23523 PMC7542609

[15] Aldskogius H. Microglia in Neuroregeneration. 2001;46:40–6. 10.1002/jemt.1119 11526956

[16] Neumann H, Kotter MR, Franklin RJM. Debris clearance by microglia: an essential link between degeneration and regeneration. Brain: a journal of neurology. 2009;132:288–95. 18567623 10.1093/brain/awn109 PMC2640215

[17] Guan P, Fan L, Zhu Z, Yang Q, Kang X, Li J, M2 microglia-derived exosome-loaded electroconductive hydrogel for enhancing neurological recovery after spinal cord injury. J Nanobiotechnol. 2024;22:8. 10.1186/s12951-023-02255-w PMC10763283 38167113

[18] Zhao S, Umpierre AD, Wu L-J. Tuning neural circuits and behaviors by microglia in the adult brain. Trends in Neurosciences. 2024;47:181–94. 38245380 10.1016/j.tins.2023.12.003 PMC10939815

[19] Aguzzi A, Barres B a, Bennett ML. Microglia: Scapegoat, Saboteur or Something Else ? Inflammation. 2013;339:156–62. 10.1126/science.1227901 PMC4431634 23307732

[20] Ramlackhansingh AF, Brooks DJ, Greenwood RJ, Bose SK, Turkheimer FE, Kinnunen KM, Inflammation after trauma: microglial activation and traumatic brain injury. Annals of neurology. 2011;70:374–83. 21710619 10.1002/ana.22455

[21] Nizamutdinov D, Shapiro LA. Overview of traumatic brain injury: An immunological context. Brain Sciences. 2017;7. 10.3390/brainsci7010011 PMC5297300 28124982

[22] Clark KC, Josephson A, Benusa SD, Hartley RK, Baer M, Thummala S, Compromised axon initial segment integrity in EAE is preceded by microglial reactivity and contact. Glia. 2016;64:1190–209. 27100937 10.1002/glia.22991

[23] Eyo UB, Wu LJ. Bidirectional microglia-neuron communication in the healthy brain. Neural Plasticity. 2013;2013. 10.1155/2013/456857 PMC3775394 24078884

[24] Eyo UB, Gu N, De S, Dong H, Richardson JR, Wu L-J. Modulation of Microglial Process Convergence Toward Neuronal Dendrites by Extracellular Calcium. Journal of Neuroscience. 2015;35:2417–22. 25673836 10.1523/JNEUROSCI.3279-14.2015 PMC4323526

[25] Eyo UB, Bispo A, Liu J, Sabu S, Wu R, Dibona VL, The GluN2A Subunit Regulates Neuronal NMDA receptor-Induced Microglia-Neuron Physical Interactions. Scientific Reports. 2018; 10.1038/s41598-018-19205-4 PMC5770428 29339791

[26] Gorse KM, Lafrenaye AD. The importance of inter-species variation in traumatic brain injury-induced alterations of microglial-axonal interactions. Frontiers in Neurology. 2018;9:778. 30294296 10.3389/fneur.2018.00778 PMC6158363

[27] Lafrenaye AD, Todani M, Walker SA, Povlishock JT. Microglia processes associate with diffusely injured axons following mild traumatic brain injury in the micro pig. Journal of Neuroinflammation. 2015;12:186. 26438203 10.1186/s12974-015-0405-6 PMC4595283

[28] Lafrenaye A. Physical interactions between activated microglia and injured axons: do all contacts lead to phagocytosis? Neural Regeneration Research. 2016;11:538. 27212901 10.4103/1673-5374.180726 PMC4870897

[29] HuY, TaoW. Current perspectives on microglia-neuron communication in the central nervous system: Direct and indirect modes of interaction. Journal of Advanced Research [Internet]. 2024 [cited 2024 May 1]; Available from: https://www.sciencedirect.com/science/article/pii/S209012322400006710.1016/j.jare.2024.01.00638195039

[30] Benusa SD, Lafrenaye AD. Microglial process convergence on axonal segments in health and disease. Neuroimmunology and Neuroinflammation. 2020;7:23–39. 34007863 10.20517/2347-8659.2019.28 PMC8128155

[31] Mesfin FB, Gupta N, Hays Shapshak A, Taylor RS. Diffuse Axonal Injury. StatPearls [Internet]. Treasure Island (FL): StatPearls Publishing; 2023 [cited 2023 May 10]. Available from: http://www.ncbi.nlm.nih.gov/books/NBK448102/

[32] Graham NSN, Jolly A, Zimmerman K, Bourke NJ, Scott G, Cole JH, Diffuse axonal injury predicts neurodegeneration after moderate–severe traumatic brain injury. Brain. 2020;143:3685–98. 33099608 10.1093/brain/awaa316

[33] Frati A, Cerretani D, Fiaschi A, Frati P, Gatto V, La Russa R, Diffuse Axonal Injury and Oxidative Stress: A Comprehensive Review. International Journal of Molecular Sciences. 2017;18:2600. 29207487 10.3390/ijms18122600 PMC5751203

[34] Büki a Povlishock JT. All roads lead to disconnection?--Traumatic axonal injury revisited. Acta neurochirurgica. 2006;148:181–93; discussion 193–4. 10.1007/s00701-005-0674-4 16362181

[35] Smith DH, Meaney DF, Shull WH. Diffuse axonal injury in head trauma. J Head Trauma Rehabil. 2003;18:307–16. 16222127 10.1097/00001199-200307000-00003

[36] Chen Q, Chen X, Xu L, Zhang R, Li Z, Yue X, Traumatic axonal injury: neuropathological features, postmortem diagnostic methods, and strategies. Forensic Sci Med Pathol. 2022;18:530–44. 36117238 10.1007/s12024-022-00522-0

[37] Maxwell WL, Watt C, Graham DI, Gennarelli TA. Ultrastructural evidence of axonal shearing as a result of lateral acceleration of the head in non-human primates. Acta Neuropathol. 1993;86:136–44. 7692693 10.1007/BF00334880

[38] Povlishock JT. Pathobiology of traumatically induced axonal injury in animals and man. Annals of Emergency Medicine. 1993;22:980–6. 8503536 10.1016/s0196-0644(05)82738-6

[39] Christman CW, Grady MS, Walker SA, Holloway KL, Povlishock JT. Ultrastructural studies of diffuse axonal injury in humans. Journal of neurotrauma. 1994;11:173–86. 7523685 10.1089/neu.1994.11.173

[40] Stone JR, Okonkwo DO, Dialo AO, Rubin DG, Mutlu LK, Povlishock JT, Impaired axonal transport and altered axolemmal permeability occur in distinct populations of damaged axons following traumatic brain injury. Experimental neurology. 2004;190:59–69. 15473980 10.1016/j.expneurol.2004.05.022

[41] Gentleman SM, Nash MJ, Sweeting CJ, Graham DI, Roberts GW. β-Amyloid precursor protein (βAPP) as a marker for axonal injury after head injury. Neuroscience Letters. 1993;160:139–44. 8247344 10.1016/0304-3940(93)90398-5

[42] Sherriff FE, Bridges LR, Sivaloganathan S. Early detection of axonal injury after human head trauma using immunocytochemistry for beta-amyloid precursor protein. Acta neuropathologica. 1994;87:55–62. 8140894 10.1007/BF00386254

[43] DiLeonardi AM, Huh JW, Raghupathi R. Impaired axonal transport and neurofilament compaction occur in separate populations of injured axons following diffuse brain injury in the immature rat. Brain Research. 2009;1263:174–82. 19368848 10.1016/j.brainres.2009.01.021 PMC2696174

[44] Bramlett HM, Kraydieh S, Green EJ, Dietrich WD. Temporal and regional patterns of axonal damage following traumatic brain injury: A beta-amyloid precursor protein immunocytochemical study in rats. Journal of Neuropathology and Experimental Neurology. 1997;56:1132–41. 9329457 10.1097/00005072-199710000-00007

[45] Chen X-H, Siman R, Iwata A, Meaney DF, Trojanowski JQ, Smith DH. Long-term accumulation of amyloid-beta, beta-secretase, presenilin-1, and caspase-3 in damaged axons following brain trauma. The American journal of pathology. 2004;165:357–71. 15277212 10.1016/s0002-9440(10)63303-2 PMC1618579

[46] Johnson VE, Stewart W, Smith DH. Widespread tau and amyloid-beta pathology many years after a single traumatic brain injury in humans. Brain Pathology. 2012;22:142–9. 21714827 10.1111/j.1750-3639.2011.00513.x PMC3979351

[47] Johnson VE, Stewart W, Smith DH. Axonal pathology in traumatic brain injury. Experimental Neurology. 2013;246:35–43. 22285252 10.1016/j.expneurol.2012.01.013 PMC3979341

[48] Morganti-Kossmann MC, Satgunaseelan L, Bye N, Kossmann T. Modulation of immune response by head injury. Injury. 2007;38:1392–400. 18048036 10.1016/j.injury.2007.10.005

[49] Kelley BJ, Lifshitz J, Povlishock JT. Neuroinflammatory responses after experimental diffuse traumatic brain injury. J Neuropathol Exp Neurol. 2007;66:989–1001. 17984681 10.1097/NEN.0b013e3181588245

[50] Zhou Y, Lui YW, Zuo X-N, Milham MP, Reaume J, Grossman RI, Characterization of thalamocortical association using amplitude and connectivity of fMRI in mild traumatic brain injury. J Magn Reson Imaging. 2014;39:1558–68. 24014176 10.1002/jmri.24310 PMC3872273

[51] Mannix RC, Whalen MJ. Traumatic brain injury, microglia, and Beta amyloid. International journal of Alzheimer’s disease. 2012;2012:608732. 10.1155/2012/608732 PMC3359797 22666622

[52] Smith C. Review: The long-term consequences of microglial activation following acute traumatic brain injury. Neuropathology and Applied Neurobiology. 2013;39:35–44. 23206160 10.1111/nan.12006

[53] Ransohoff RM, Perry VH. Microglial physiology: unique stimuli, specialized responses. Annual review of immunology. 2009;27:119–45. 10.1146/annurev.immunol.021908.132528 19302036

[54] Karve IP, Taylor JM, Crack PJ. The contribution of astrocytes and microglia to traumatic brain injury. British Journal of Pharmacology. 2015;n/a-n/a. 10.1111/bph.13125 PMC4742296 25752446

[55] Tay TL, Hagemeyer N, Prinz M. The force awakens: Insights into the origin and formation of microglia. Current Opinion in Neurobiology. 2016;39:30–7. 27107946 10.1016/j.conb.2016.04.003

[56] Loane DJ, Kumar A. Microglia in the TBI brain: The good, the bad, and the dysregulated. Experimental Neurology. 2016;275:316–27. 26342753 10.1016/j.expneurol.2015.08.018 PMC4689601

[57] Grovola MR, von Reyn C, Loane DJ, Cullen DK. Understanding microglial responses in large animal models of traumatic brain injury: an underutilized resource for preclinical and translational research. J Neuroinflammation. 2023;20:67. 36894951 10.1186/s12974-023-02730-z PMC9999644

[58] Ronzano R, Roux T, Thetiot M, Aigrot MS, Richard L, Lejeune FX, Microglia-neuron interaction at nodes of Ranvier depends on neuronal activity through potassium release and contributes to remyelination. Nat Commun. 2021;12:5219. 34471138 10.1038/s41467-021-25486-7 PMC8410814

[59] Wake H, Moorhouse AJ, Jinno S, Kohsaka S, Nabekura J. Resting microglia directly monitor the functional state of synapses in vivo and determine the fate of ischemic terminals. The Journal of neuroscience: the official journal of the Society for Neuroscience. 2009;29:3974–80. 19339593 10.1523/JNEUROSCI.4363-08.2009 PMC6665392

[60] Uweru JO, Eyo UB. A Decade of Diverse Microglial-Neuronal Physical Interactions in the Brain (2008–2018). Neurosci Lett. 2019;698:33–8. 30625349 10.1016/j.neulet.2019.01.001 PMC6435396

[61] Weber MD, McKim DB, Niraula A, Witcher KG, Yin W, Sobol CG, The Influence of Microglial Elimination and Repopulation on Stress Sensitization Induced by Repeated Social Defeat. Biological Psychiatry. 2019;85:667–78. 30527629 10.1016/j.biopsych.2018.10.009 PMC6440809

[62] d’Avila JC, Lam TI, Bingham D, Shi J, Won S, Kauppinen TM, Microglial activation induced by brain trauma is suppressed by post-injury treatment with a PARP inhibitor. Journal of Neuroinflammation. 2012;9:31. 22335939 10.1186/1742-2094-9-31 PMC3298794

[63] Siopi E, Llufriu-Dabén G, Fanucchi F, Plotkine M, Marchand-Leroux C, Jafarian-Tehrani M. Evaluation of late cognitive impairment and anxiety states following traumatic brain injury in mice: The effect of minocycline. Neuroscience Letters. 2012;511:110–5. 22314279 10.1016/j.neulet.2012.01.051

[64] Henry RJ, Ritzel RM, Barrett JP, Doran SJ, Jiao Y, Leach JB, Microglial depletion with CSF1R inhibitor during chronic phase of experimental traumatic brain injury reduces neurodegeneration and neurological deficits. bioRxiv. 2019;791871. 10.1523/JNEUROSCI.2402-19.2020 PMC7117897 32094203

[65] Swiatkowski P, Murugan M, Eyo UB, Wang Y, Rangaraju S, Oh SB, Activation of microglial P2Y12 receptor is required for outward potassium currents in response to neuronal injury. Neuroscience. 2016;318:22–33. 26791526 10.1016/j.neuroscience.2016.01.008 PMC4753827

[66] Dissing-Olesen L, LeDue JM, Rungta RL, Hefendehl JK, Choi HB, MacVicar BA. Activation of Neuronal NMDA Receptors Triggers Transient ATP-Mediated Microglial Process Outgrowth. Journal of Neuroscience. 2014;34:10511–27. 25100586 10.1523/JNEUROSCI.0405-14.2014 PMC6802598

[67] Eyo UB, Peng J, Swiatkowski P, Mukherjee A, Bispo A, Wu L-J. Neuronal hyperactivity recruits microglial processes via neuronal NMDA receptors and microglial P2Y12 receptors after status epilepticus. The Journal of neuroscience: the official journal of the Society for Neuroscience. 2014;34:10528–40. 25100587 10.1523/JNEUROSCI.0416-14.2014 PMC4200107

[68] Franco ECS, Cardoso MM, Gouvêia A, Pereira A, Gomes-Leal W. Modulation of microglial activation enhances neuroprotection and functional recovery derived from bone marrow mononuclear cell transplantation after cortical ischemia. Neuroscience Research. 2012;73:122–32. 22465414 10.1016/j.neures.2012.03.006

[69] Hanlon LA, Raghupathi R, Huh JW. Depletion of microglia immediately following traumatic brain injury in the pediatric rat: Implications for cellular and behavioral pathology. Experimental Neurology. 2019;316:39–51. 30980832 10.1016/j.expneurol.2019.04.004 PMC6544393

[70] Wang C, Ji Y, Zhang H, Ye Y, Zhang G, Zhang S, Increased level of exosomal miR-20b-5p derived from hypothermia-treated microglia promotes neurite outgrowth and synapse recovery after traumatic brain injury. Neurobiology of Disease. 2023;179:106042. 36804284 10.1016/j.nbd.2023.106042

[71] Batchelor PE, Porritt MJ, Martinello P, Parish CL, Liberatore GT, Donnan G a, Macrophages and Microglia Produce Local Trophic Gradients That Stimulate Axonal Sprouting Toward but Not beyond the Wound Edge. Molecular and cellular neurosciences. 2002;21:436–53. 12498785 10.1006/mcne.2002.1185

[72] Parkhurst CN, Yang G, Ninan I, Savas JN, Yates JR, Lafaille JJ, Microglia promote learning-dependent synapse formation through brain-derived neurotrophic factor. Cell. 2013;155:1596–609. 24360280 10.1016/j.cell.2013.11.030 PMC4033691

[73] Dougherty KD, Dreyfus CF, Black IB. Brain-derived neurotrophic factor in astrocytes, oligodendrocytes, and microglia/macrophages after spinal cord injury. Neurobiology of disease. 2000;7:574–85. 11114257 10.1006/nbdi.2000.0318

[74] Liu YU, Ying Y, Li Y, Eyo UB, Chen T, Zheng J, Neuronal network activity controls microglial process surveillance in awake mice via norepinephrine signaling. Nat Neurosci. 2019;22:1771–81. 31636449 10.1038/s41593-019-0511-3 PMC6858573

[75] Cao K, Qiu L, Lu X, Wu W, Hu Y, Cui Z, Microglia modulate general anesthesia through P2Y12 receptor. Current Biology. 2023;33:2187–2200.e6. 37167975 10.1016/j.cub.2023.04.047

[76] Schirmer L, Merkler D, König FB, Brück W, Stadelmann C. Neuroaxonal regeneration is more pronounced in early multiple sclerosis than in traumatic brain injury lesions. Brain Pathology. 2013;23:2–12. 22612622 10.1111/j.1750-3639.2012.00608.x PMC8057635

[77] Oehmichen M, Theuerkauf I, Meissner C. Is traumatic axonal injury (AI) associated with an early microglial activation? Application of a double-labeling technique for simultaneous detection of microglia and AI. Acta neuropathologica. 1999;97:491–4. 10334486 10.1007/s004010051018

[78] Ryu J, Horkayne-Szakaly I, Xu L, Pletnikova O, Leri F, Eberhart C, The problem of axonal injury in the brains of veterans with histories of blast exposure. Acta Neuropathologica Communications. 2014;2:1–14. 25422066 10.1186/s40478-014-0153-3 PMC4260204

[79] Biegon A. Considering Biological Sex in Traumatic Brain Injury. Frontiers in Neurology. 2021;12:576366. 33643182 10.3389/fneur.2021.576366 PMC7902907

[80] Song H, Tomasevich A, Paolini A, Browne KD, Wofford KL, Kelley B, Sex differences in the extent of acute axonal pathologies after experimental concussion. Acta Neuropathol. 2024;147:79. 38705966 10.1007/s00401-024-02735-9 PMC11070329

[81] Yanguas-Casás N. Physiological sex differences in microglia and their relevance in neurological disorders. Neuroimmunology and Neuroinflammation. 2020;7:13–22.

[82] Martinez-Muniz GA, Wood SK. Special Section on Sexual Dimorphism in Neuroimmune Cells Sex Differences in the Inflammatory Consequences of Stress: Implications for Pharmacotherapy. THE JOURNAL OF PHARMACOLOGY AND EXPERIMENTAL THERAPEUTICS J Pharmacol Exp Ther. 2020;375:161–74. 10.1124/jpet.120.266205 PMC7569308 32759370

[83] VanRyzin JW, Pickett LA, McCarthy MM. Microglia: Driving critical periods and sexual differentiation of the brain. Developmental Neurobiology. 2018;78:580–92. 29243403 10.1002/dneu.22569 PMC5980665

[84] Schwarz JM, Sholar PW, Bilbo SD. Sex differences in microglial colonization of the developing rat brain. Journal of Neurochemistry. 2012;120:948–63. 22182318 10.1111/j.1471-4159.2011.07630.x PMC3296888

[85] Doran SJ, Ritzel RM, Glaser EP, Henry RJ, Faden AI, Loane DJ. Sex differences in acute neuroinflammation after experimental traumatic brain injury are mediated by infiltrating myeloid cells. Journal of Neurotrauma. 2019;36:1040–53. 30259790 10.1089/neu.2018.6019 PMC6444913

